# Multi-Head Attention-Based Framework with Residual Network for Human Action Recognition

**DOI:** 10.3390/s25092930

**Published:** 2025-05-06

**Authors:** Basheer Al-Tawil, Magnus Jung, Thorsten Hempel, Ayoub Al-Hamadi

**Affiliations:** Neuro-Information Technology, Otto-von-Guericke-University Magdeburg, 39106 Magdeburg, Germany; magnus.jung@ovgu.de (M.J.); thorsten.hempel@ovgu.de (T.H.)

**Keywords:** human action recognition, residual networks, multi-head attention, Bi-LSTM, UCF-101, spatial feature, temporal modeling

## Abstract

Human action recognition (HAR) is essential for understanding and classifying human movements. It is widely used in real-life applications such as human–computer interaction and assistive robotics. However, recognizing patterns across different temporal scales remains challenging. Traditional methods struggle with complex timing patterns, intra-class variability, and inter-class similarities, leading to misclassifications. In this paper, we propose a deep learning framework for efficient and robust HAR. It integrates residual networks (ResNet-18) for spatial feature extraction and Bi-LSTM for temporal feature extraction. A multi-head attention mechanism enhances the prioritization of crucial motion details. Additionally, we introduce a motion-based frame selection strategy utilizing optical flow to reduce redundancy and enhance efficiency. This ensures accurate, real-time recognition of both simple and complex actions. We evaluate the framework on the UCF-101 dataset, achieving a 96.60% accuracy, demonstrating competitive performance against state-of-the-art approaches. Moreover, the framework operates at 222 frames per second (FPS), achieving an optimal balance between recognition performance and computational efficiency. The proposed framework was also deployed and tested on a mobile service robot, TIAGo, validating its real-time applicability in real-world scenarios. It effectively models human actions while minimizing frame dependency, making it well-suited for real-time applications.

## 1. Introduction

Human action recognition (HAR) from video data has emerged as a transformative area in computer vision [1,2,3,4,5,6,7,8]. It enables machines to understand human behavior in video and predict ongoing actions, reshaping how technology interacts with the physical world. HAR has numerous real-world applications, including surveillance, healthcare, smart devices, and robotics [3,4,5]. Unlike static image recognition, which focuses on individual frames, video-based HAR integrates both spatial and temporal cues. This allows systems to capture the evolving nature of human actions.

Early HAR approaches used handcrafted features such as optical flow and silhouette-based representations, which were analyzed using models such as Hidden Markov Models (HMMs) and Support Vector Machines (SVMs) [1]. However, these methods struggled to capture complex temporal dynamics and generalize to real-world conditions. With the advance of deep learning, convolutional neural networks (CNNs) began to enable powerful spatial feature extraction across video frames [9]. Recurrent models such as long short-term memory (LSTM) networks and Gated Recurrent Units (GRUs) then enabled better modeling of motion and context over time [10]. However, many of these models are large and computationally expensive, limiting their use on edge devices such as mobile robots.

Real-time HAR remains challenging due to several factors: significant intra-class variation (e.g., different walking styles), subtle inter-class differences (e.g., running vs. jogging), and environmental variations such as occlusion or lighting [3]. Furthermore, training large-scale deep models on thousands of video sequences requires considerable computation [11]. These challenges are even more pronounced when deploying HAR on resource-constrained systems.

To address these issues, we propose an efficient HAR framework that balances accuracy and computational cost, making it suitable for real-time deployment in robotic platforms and edge devices. Our model built on two design strategies:

- **Motion-Aware Frame Selection:** We prioritize high-motion frames using motion scores derived from optical flow, reducing redundancy and enhancing relevant temporal dynamics.
- **Multi-Head Attention Integration:** A multi-head attention mechanism refines the Bi-LSTM output by emphasizing the most informative spatiotemporal features across the sequence.

**Figure 1:**
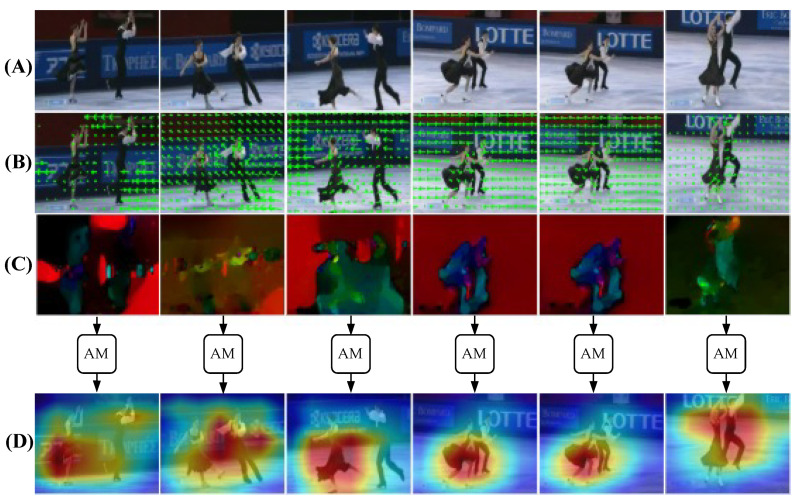
Visualization of the motion-aware frame selection strategy, combining optical flow analysis and attention-based interpretation for human action recognition: (**A**) original frames, (**B**) motion vectors; arrows show movement direction. (**C**) Flow magnitudes and directions—red indicates rightward motion, blue indicates leftward motion, yellow represents upward motion, and green represents downward motion, (**D**) Grad-CAM heatmap highlighting class-relevant motion regions after processing through the framework. Blue, yellow, and red represent features with minimal, medium, and high significance, respectively, as perceived by the model. The video sample is randomly selected from the UCF-101 dataset [13].

**Figure 2:**
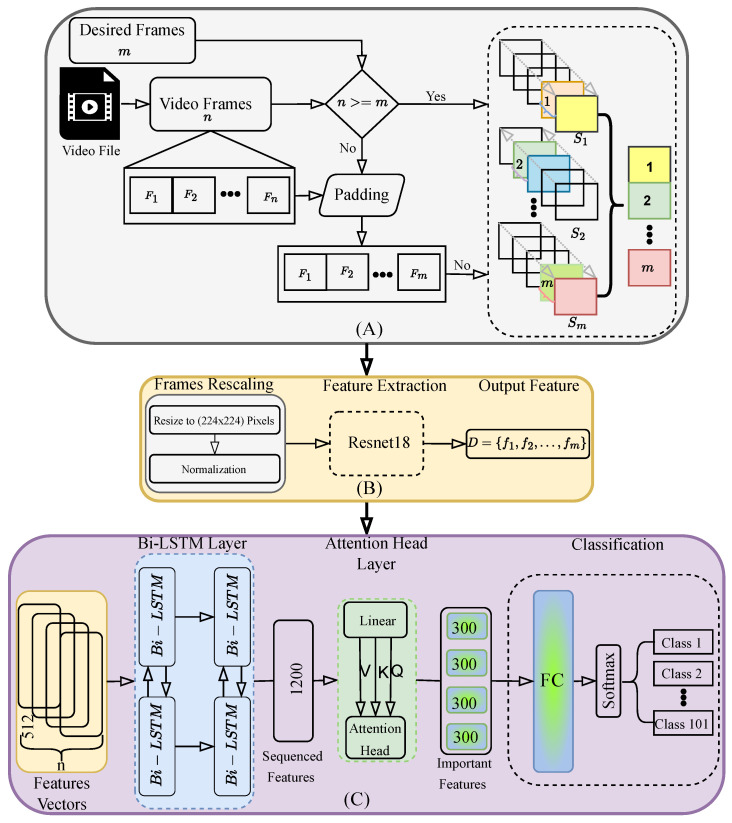
Overview of the proposed human action recognition framework. The pipeline consists of (**A**) frame selection—selecting the most relevant frames from input videos using a scoring strategy; (**B**) feature extraction—applying data augmentation and extracting spatial features using a ResNet-18 model; and (**C**) classification—capturing temporal dependencies using a Bi-LSTM and refining feature relations using a multi-head attention mechanism, followed by action classification using a fully connected layer and a softmax function.

We adopt ResNet-18 for spatial feature extraction due to its balance between efficiency and representation quality. A bidirectional LSTM captures temporal dependencies in both directions, improving generalization, especially on smaller datasets. The attention mechanism further improves the model’s ability to highlight key motion cues across time. Experiments on the UCF-101 benchmark show that our framework achieves 96.60% accuracy and runs at 222 frames per second (FPS). It outperforms several state-of-the-art models in both performance and speed. To demonstrate its practical use, we deployed the model on the mobile service robot TIAGo [12]. It achieved reliable action recognition in real-time robotic scenarios. Figure 1 shows a visualization summary of the framework steps, while Figure 2 presents the complete architecture of the framework. All steps will be described in detail in the following sections.

The rest of the paper is structured as follows: Section 2 reviews related HAR models and their efficiency limitations. Section 3 explains the proposed framework in detail. Section 4 outlines the training and evaluation protocols. Section 5 presents the results and comparisons. Finally, Section 6 concludes with our key findings and future work directions. The source code will be publicly available at https://github.com/basheeraltawil/HAR-ResNet-BiLSTM-Attention.git (accessed on 25 April 2025).

## 2. Related Work

HAR has evolved significantly over the years, driven by advances in computer vision and deep learning [2,6,14]. The field has evolved from early hand-crafted features to more sophisticated, deep-learning-based approaches that enable better recognition of complex actions in video sequences [1].

### 2.1. Early Methods in HAR

Early methods in HAR relied on statistical models and machine learning techniques, which laid the foundation for more sophisticated approaches. These techniques focused primarily on capturing spatial or temporal features but struggled to model complex motion dynamics and long-range temporal dependencies [15].

#### 2.1.1. Hidden Markov Models (HMMs)

HMMs were among the first statistical models applied to HAR, capturing temporal patterns by defining states corresponding to different stages of an action [16,17]. These models are suitable for capturing the probabilistic nature of action sequences and have been used for gesture recognition [16,17]. However, HMMs have limitations in modeling complex temporal relationships and long-range dependencies. This makes them less effective for dynamic and real-world scenarios that require detailed and long-term understanding of actions [18]. As the need for more robust temporal modeling grew, deep learning models began to emerge. Unlike HMMs, these models learn temporal dependencies directly from the data, leading to significant improvements [19,20].

#### 2.1.2. Support Vector Machines (SVMs)

SVMs have been widely used in HAR to classify actions based on spatial features extracted from individual video frames. They work by finding an optimal hyperplane that separates different action classes in a high-dimensional feature space [21]. When paired with features such as optical flow, SVMs have shown promise in action recognition. However, they struggle to capture temporal dependencies across video frames. This makes them less suitable for actions that involve continuous motion, such as running or jumping [14,22]. These early models laid the foundation for HAR. However, their inability to handle complex temporal relationships and motion dynamics led to the development of more advanced deep-learning-based architectures [14,18].

### 2.2. Deep-Learning-Based Methods

Deep-learning-based approaches have revolutionized Human Activity Recognition (HAR). They effectively combine spatial and temporal information. These models use convolutional neural networks (CNNs), recurrent neural networks (RNNs), and long short-term memory (LSTM) networks. They have advanced the field by learning from large datasets. These methods also overcome the limitations of previous approaches [23].

#### 2.2.1. Convolutional Neural Networks (CNNs) for Spatial Feature Extraction

CNNs have brought significant progress to HAR by enabling automatic extraction of spatial features directly from video frames [24,25]. The two-stream CNN architecture proposed by Simonyan and Zisserman [26] used both RGB frames for spatial information and optical flow for motion-related features. This two-stream approach achieved significant improvements in action recognition by capturing both visual content and motion dynamics. This approach proved effective for static spatial features, but had limitations - it processed frames independently and lacked the ability to capture temporal dependencies between frames [7,16,27]. State-of-the-art models, such as SlowFast Networks [28], use a two-stream architecture. They combine a slow stream for spatial features and a fast stream for motion. This approach efficiently captures both spatial and temporal information. These models showed significant improvements in action recognition performance on large-scale benchmarks such as Kinetics and UCF-101.

#### 2.2.2. RNNs and LSTM Networks for Temporal Modeling

RNNs and LSTM networks are critical for modeling temporal dependencies in HAR. RNNs process video frames sequentially. They capture the temporal evolution of actions [10,29]. LSTM networks, a more advanced form of RNNs, are designed to handle long-term dependencies. This makes them ideal for recognizing actions that require understanding motion over time [30,31]. Recent advances in HAR have integrated LSTM networks with CNNs. This has led to hybrid architectures such as long-term recurrent convolutional networks (LRCNs) [31]. These networks combine CNNs for spatial feature extraction and LSTM networks for modeling temporal relationships. This combination has significantly improved recognition performance. It is particularly effective for actions with complex temporal dependencies. However, these methods still face challenges in handling large, high-dimensional data. To address this, models such as Temporal Convolutional Networks (TCNs) [32] and 3D CNNs [33] have been proposed. These models directly capture spatiotemporal dependencies by modeling both spatial and temporal features simultaneously. While these methods show strong performance, their computational complexity makes them less suitable for real-time applications [34].

### 2.3. Attention-Based Action Recognition

Attention mechanisms have become important in improving human action recognition (HAR) models. They help the model focus on the most relevant parts of the video by giving more weight to important frames and features [6,35]. This makes it easier to recognize actions accurately, especially when there is considerable motion or background noise. In recent years, advanced attention mechanisms have been developed to better handle complex visual scenarios. Query-guided attention [36] improves occlusion handling by dynamically updating target features and guiding focus through cross-fusion layers. Capsule attention [37] enhances feature representation using self-weighted capsule vectors and spectral–spatial attention, while consistent representation mining [38] leverages sparse multi-head attention to extract invariant features across multi-drone views.

These developments align with the trend in HAR research toward more adaptive and motion-aware attention strategies. The Action Transformer [39] uses spatiotemporal attention to model relationships across frames and regions, enabling effective recognition of complex actions. Similarly, STS-ALSTM (Spatiotemporal Sequence Attention LSTM) [40] integrates attention with LSTM networks to dynamically adjust frame weights, improving adaptation to varying action contexts. The Fluxformer [41] advances attention-based HAR by combining optical flow and RGB features through a duplex attention mechanism, using spatial clustering and flow-guided tokenization to preserve critical details. These advances highlight the value of integrating attention with motion cues, such as optical flow, for robust spatiotemporal modeling in HAR [42].

## 3. Methodology

This section outlines the methodology of the proposed framework, which integrates spatial and temporal modeling with an attention mechanism, as shown in Figure 2. The workflow includes frame sampling and sequence generation, feature extraction, and model structuring.

### 3.1. Video Preprocessing

The following preprocessing steps, shown in Figure 2 part (A) and Algorithm 1, are explained below and were used to prepare video files as input for our model.

Frame Sampling: For each video, the total number of frames is denoted by *n*, and the target number of frames is set to *m*. If n<m, additional frames are added to the sequence by randomly duplicating frames from the original sequence while maintaining the chronological order of the frames. This technique ensures temporal consistency and helps preserve the dynamic features of the video, allowing the model to better capture subtle changes in motion that are critical for human action recognition [43].
**Algorithm 1** Frame Selection Process1:**Input:** Video file with *n* frames, desired frames *m*2:**Output:** Selected high score frames3:Extract frames F={1,2,…,n} from video4:**if** n<m **then**5:      Apply padding to increase frame count to *m*6:**end if**7:Divide frames into *m* sequences $S_{1},S_{2},\ldots ,S_{m}$8:**for** each sequence $S_{i}$ **do**9:      Compute score for each frame in $S_{i}$10:     Select the highest scoring frame from $S_{i}$11:**end for**12:Return the selected frames(1,2,…, *m*)

Sequence Generation: To capture different temporal dynamics and improve generalization, video frames are divided into $S_{1},S_{2},\ldots ,S_{m}$ equally spaced sub-sequences. Each subsequence has a length of $\frac{n}{m}$. This method provides the framework with a wide range of temporal action patterns while maintaining efficiency [44].

Motion-Based Frame Selection: To reduce redundancy, frames with the most significant motion are selected using the Farneback method [45], which uses motion scores derived from optical flow. This also allows better integration into time-critical (real-time) applications, since not all frames need to be processed based on the selection of relevant frames. Frames without additional information can be skipped without loss of estimation accuracy. The motion score ($M_{s}$) is calculated for each pair of frames based on the displacement of pixels between consecutive frames, as given by Equation (1):

(1) $$M_{s}=\sum_{x=1}^{W}\sum_{y=1}^{H}\sqrt{u{\left(x,y\right)}^{2}+v{\left(x,y\right)}^{2}},$$

where *W* and *H* are the width and height of the frame and ((u(x,y),v(x,y)) are the horizontal and vertical pixel shifts between consecutive frames. Frames with the highest motion scores are selected to ensure that the most dynamic parts of the video are preserved [46].

As visualized in Figure 1, optical flow analysis serves as an effective mechanism for identifying frames with significant motion content in human action recognition. The model leverages optical flow to quantify movement between consecutive frames, enabling the selection of key frames that best represent the action sequence. By measuring motion magnitude and direction, the system can identify frames containing essential action components while filtering out redundant or static frames, resulting in a more efficient and representative subset of frames for action classification.

**Figure 3:**
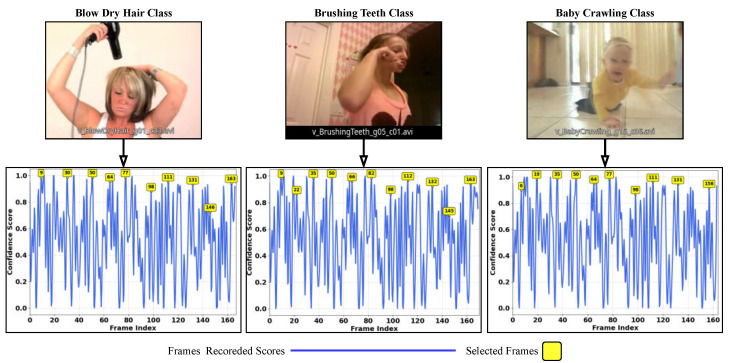
Three randomly selected classes from the UCF-101 test dataset [13], illustrating the frame selection process based on confidence values. The selected frames correspond to those with the highest confidence, with their indices given.

Frame Resizing and Normalization: The selected frames are resized to 224×224 pixels and normalized using the standard deviation values derived from the ImageNet dataset to meet the input requirements of the ResNet-18 model. As in Equation (2), μ and σ represent the channel-wise mean and standard deviation for the normalization, which is most commonly used in the video frame normalization process. Finally, to illustrate how frames are selected based on score confidence, we provide a graphical demonstration. Figure 3 shows three videos from the UCF-101 dataset: hair drying, teeth brushing, and a baby crawling. The figure highlights where the frame selection occurs, based on the confidence of the score at the optimal maximum of the graph. The selection process occurs every 10 frames along the segment size, as specified in this case.

(2) $$\mu =\left[\begin{array}{c} 0.485\\ 0.456\\ 0.406 \end{array}\right]\;\mathrm{and}\;\sigma =\left[\begin{array}{c} 0.229\\ 0.224\\ 0.225 \end{array}\right]$$

### 3.2. Feature Extraction with ResNet-18

Recent studies confirm ResNet-18’s effectiveness in human action recognition, emphasizing its ability to efficiently extract meaningful features [47,48]. Based on these findings, we use ResNet-18 in our framework for spatial feature extraction. This architecture is designed to address the vanishing gradient problem through skip connections, enabling effective training while preserving spatial features. It is particularly well-suited for processing individual video frames as images [47,49].

ResNet-18 strikes an optimal balance between performance and computational efficiency, making it ideal for mobile robotics applications. Although deeper variants like ResNet-50 or ResNet-101 could offer slightly higher accuracy, they incur significantly greater computational overhead. ResNet-18 is efficient in real-time applications, providing faster inference times and reduced memory usage due to fewer parameters [49]. Its simplicity also mitigates overfitting, making it a robust choice for human action recognition tasks.

The feature extraction process is illustrated in Figure 2 part (B), where preprocessed frames are input into ResNet-18. The resulting features are stored sequentially as $D=\{f_{1},f_{2},\ldots ,f_{m}\}$, with each frame yielding a 512-dimensional feature vector *f* by removing the final classification layer.

### 3.3. Model Structuring

As shown in Figure 2 part (C), we integrate the Bi-LSTM network with a multi-head attention mechanism to improve the HAR approach and make it more adaptive to different weights of classes. First, we take the features obtained from ResNet-18 as shown in Figure 2. Then, they are processed by the Bi-LSTM hidden states *h*; see Equation (3). It analyzes sequences in both forward ($h_{t}^{f}$) and backward ($h_{t}^{b}$) directions, allowing the model to understand the motion progression in a sequential manner to extract temporal features. The final output for each time step is given by concatenating the hidden states from both directions.

(3) $$h_{t}^{LSTM}=\left[h_{t}^{f},h_{t}^{b}\right]$$

After the Bi-LSTM processing, a multi-head attention mechanism is integrated to capture the important sequence for classification. The transition from Bi-LSTM to multi-head attention is facilitated by using the hidden states $h_{t}^{LSTM}$ as inputs to the attention mechanism. Then, the attention scores for each frame are computed using the scaled dot product attention formula; see Equation (4).

(4) $$\alpha_{ij}=\frac{\exp\left(e_{ij}\right)}{\sum_{k=1}^{H}\exp\left(e_{ik}\right)},\;e_{ij}=\frac{Q_{i}K_{j}^{T}}{\sqrt{d_{k}}},$$

where *H*, *Q*, *K*, $d_{k}$, *i*, and *j* represent the number of attention heads, the queries generated from the Bi-LSTM output, the keys derived from the Bi-LSTM output, the dimensionality of the key vectors, the current time step (query), and all other time steps (keys and values) used to compute the attention for time step *i*. The final context vector $c_{i}$ for each input can be represented as in Equation (5).

(5) $$c_{i}=\sum_{j=1}^{T}\alpha_{ij}V_{j},$$

where *V* represents the values corresponding to the keys. This context vector synthesizes the most informative frames, allowing the model to focus on critical moments for action recognition. For temporal modeling, we combine Bi-LSTM with a multi-head attention mechanism. While Bi-LSTM captures the sequential structure of actions, attention helps highlight important features across the entire sequence, improving the model’s focus and overall performance.

**Figure 4:**
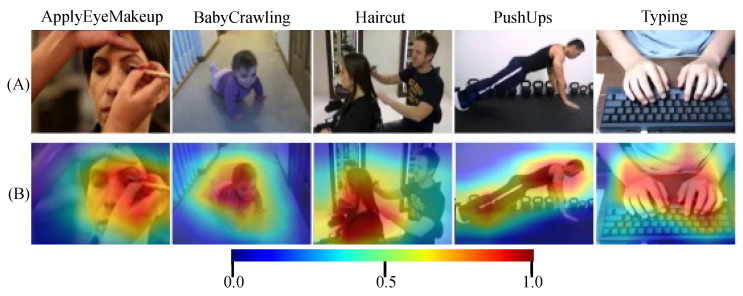
Attention visualization across five UCF-101 [13] action classes. Row (**A**) shows original video frames, while row (**B**) displays attention heatmaps where blue indicates minimal focus, and yellow/red highlights regions of high importance for action recognition.

For further clarification, we selected 5 different classes to visualize the attention heatmap across them as shown in Figure 4. The purpose of this visualization is to show how the framework generalizes across diverse action types. The framework accurately identifies and focuses on action-relevant regions within each frame. The heatmap representation in row (B) reveals the attention distribution through a color gradient: blue areas receive minimal processing attention, while yellow and red regions highlight features the model deems most significant for classification. This visualization confirms that our attention mechanism successfully filters irrelevant background information while prioritizing action-defining elements specific to each class. For instance, in the Haircut class, the network focuses on the woman’s head and the man’s hand instead of considering the man’s entire body.

The training process uses the cross-entropy loss function, which is widely adopted for multi-class classification tasks. It evaluates the discrepancy between the predicted class probabilities and the true class labels and is defined as

(6) $$L_{CE}=-\sum_{i=1}^{C}y_{i}\log\left({\hat{y}}_{i}\right),$$

where *C* is the number of classes, $y_{i}$ is the ground-truth label, and ${\hat{y}}_{i}$ is the predicted probability for class *i*. In summary, we first pre-select relevant frames using optical flow from video sub-sequences. This reduces redundancy and allows the model to skip less informative frames. As a result, the classification process becomes more efficient and supports real-time performance. The selected frames are processed using ResNet-18 and Bi-LSTM. ResNet-18 extracts spatial features, while Bi-LSTM captures temporal information. The Attention Layer then prioritizes important features to support accurate classification between different activities.

## 4. Experimental Setup

This section describes the experimental setup used to evaluate the proposed framework, including the dataset description, training configurations, and evaluation metrics. We also present the results obtained under different experimental conditions. The model results were obtained on a system running Windows 10, using 20 CPUs (40 logical cores) and four Nvidia Quadro RTX 8000 GPUs. For real-world data acquisition, the raw image streams were captured using an Astra RGB camera, published via the ROS1 middleware under the topic /xtion/rgb/image_raw. These images were processed in real time by the proposed model to estimate the corresponding human action.

### 4.1. Dataset

To evaluate the effectiveness of our method, we used the UCF-101 dataset, a widely recognized benchmark for video action recognition. The dataset consists of video samples spanning 101 action categories, including activities such as sports, body movements, and object manipulation [13]. Known for its diverse set of actions and high variability within categories, the UCF-101 dataset provides a challenging benchmark for action recognition models [4,50]. For our experiments, we focused on 100 action classes, excluding the BasketballDunk category and adding them to the Basketball category. The dataset was divided into 9957 samples for training, 1657 samples for validation, and 1706 samples for testing.

### 4.2. Training Process

Features were extracted from the dataset using ResNet-18 and stored as tensors to be used as input for the model training process. The model was trained for 50 epochs using a cross-entropy loss function with a dropout rate of 0.35 and a weight decay of $1\times 10^{-4}$. The Adam optimizer was used with a training accuracy of 99.50% and a training loss of 0.0013. Table 1 summarizes the performance of the model under different configurations. Input parameters such as number of frames, batch size, attention heads, and Bi-LSTM hidden size were systematically varied. Output results were analyzed with and without the inclusion of motion score (WM and NM, respectively) for both runtime and validation accuracy.

The experiments used fixed frame counts of 20, 50, and 70 while varying other parameters. The best performance was observed with a batch size of 8, 4 attention heads, and a hidden size of 600, highlighted in light green. In these configurations, the model achieved validation accuracies of 95.80%, 96.20%, and 96.60% for 20, 50, and 70 frames, respectively, across all WM settings. In addition, the accuracy started at 96% at 35 frames and stabilized at 70 frames and beyond. These results indicate that this configuration is optimal for the model training process. To address scalability, we conducted stress tests and trained with larger batches (up to 1000 frames per sequence) and observed near-linear scalability with a GPU memory ceiling of 24 GB, suggesting practical viability for large-scale deployments.

In terms of runtime, the inclusion of motion score (WM) increased the computational time for both feature extraction and model training. For example, with the optimal configuration, the process took 55 min and 134 min (feature extraction and training) for 20 frames, 114 min and 248 min for 50 frames, and 166 min and 309 min for 70 frames.

### 4.3. Model Performance Evaluation

In this section, we evaluate the performance of our model by analyzing the distribution of attention over different frame lengths and the improvements in accuracy. We also compare the accuracies using different approaches.

#### 4.3.1. Frame-Wise Attention

**Figure 5:**
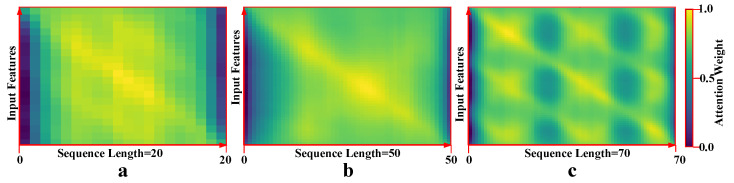
Attention weights across different sequence lengths: In the 20-frame sequence (**a**), attention is broadly distributed. In the 50-frame sequence (**b**), attention is more focused, balancing broad and periodic attention. In the 70-frame sequence (**c**), attention alternates between frames, emphasizing key moments while reducing redundancy.

We tested the model with three different sequence lengths—20, 50, and 70 frames—to understand how sequence length affects the distribution of attention weights [42,51]. For 20-frame sequences (Figure 5a), attention is broadly distributed, capturing a wide range of motion and features. In 50-frame sequences (Figure 5b), attention is slightly focused in the middle, balancing between the concentrated attention in 20-frame sequences and the periodic attention in 70-frame sequences. This middle-ground approach helps capture important motion while maintaining efficiency. For 70-frame sequences (Figure 5c), attention alternates between frames, focusing on key moments, which helps reduce redundancy but at a higher computational cost. These results show that the model adapts its attention strategy based on sequence length to efficiently capture critical temporal dynamics.

#### 4.3.2. Accuracy Analysis

**Figure 6:**
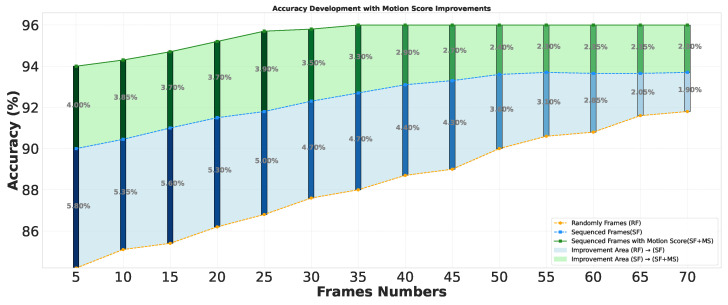
Accuracy improvement between different frame selections. The blue shaded area represents the accuracy improvement between Random Frame Selection (RF) and Sequenced Frames (SF). The green shaded area represents the improvement from Sequenced Frames (SF) to Sequenced Frames with Motion Scoring (SF + MS).

**Figure 7:**
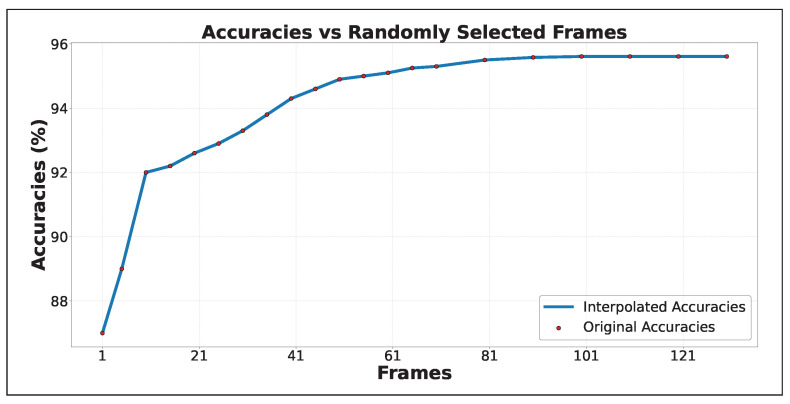
Accuracy from 1 to 130 frames. The model achieves high accuracy with few frames, making it suitable for applications requiring fast and reliable decision making.

For accuracy improvement, as shown in Figure 6, three approaches are compared: Random Frame Selection (RF), Sequenced Frames (SF), and Sequenced Frames with Motion Scoring (SF + MS). RF selects frames randomly and shows gradual improvement as more frames are included. SF selects one frame per sequence, improving faster with fewer frames. SF + MS calculates motion scores and selects the frame with the highest score, resulting in early and significant accuracy improvements. SF + MS peaks around 30 frames with 96.60% accuracy, while RF and SF improve more slowly, with SF peaking at 50 frames and RF continuing to improve gradually as more frames are processed. The differences are most noticeable before 25 frames, where SF + MS outperforms the others, highlighting the efficiency of motion scoring in capturing critical dynamics early. Furthermore, to evaluate the importance of motion scoring in achieving high accuracy, we assessed our approach using randomly selected frames that were scored early in the video. As shown in Figure 7, the model achieved 92% accuracy within the first 10 frames, resulting in high confidence values early in the video. This demonstrates that reliable class detection can be achieved with a minimal number of frames, making the approach well suited for real-time applications.

## 5. Results and Discussion

In this section, we compare our model to existing methods in terms of accuracy and efficiency. We also discuss how it reduces computational cost and improves real-time performance. Finally, we explore its potential for improving human–system interaction and situational awareness in various real-time applications.

### 5.1. Comparison with State-of-the-Art Models

Our model demonstrates high accuracy with reduced computational cost by using fewer frames compared to existing methods. Table 2 compares our approach with state-of-the-art models on the UCF-101 dataset, categorized into RNN-based, 2D/3D CNN-based, and combined deep network methods. Among the RNN-based methods, STS-ALSTM [40] and DB-LSTM [52] achieved accuracies of 92.70% and 97.00%, respectively. However, DB-LSTM requires pre-training on Kinetics-400, has 34 million parameters, and processes at least 35 frames, making it computationally expensive. In contrast, our model achieves 96.60% accuracy with only 27 million parameters and 25 frames, providing a more efficient solution.

**Table 2:** Comparison of different models on the UCF-101 dataset.

Method	#F	Modality	Pretrained	#Param	Backbone	Acc. (%)
DB-LSTM [52]	35	RGB, OF	Im.Net + K	≈34 M	LSTM	**97.00%**
CRNN [53]	30	RGB	Im.Net	≈25 M	GRU	92.30%
STS-ALSTM [40]	10	RGB, OF, SMs	Im.Net	≈135 M	LSTM	92.70%
Wang et al. [19]	64	RGB, OF	Im.Net	≈138 M	CNN, SVM	89.10%
C3D [33]	16	RGB, OF	Sp.1M	≈17 M	3D CNN	85.20%
D + BERT [54]	32	RGB, OF	K	≈95 M	3D CNN	**98.60%**
MARS [55]	64	RGB, OF	K	≈100 M	3D CNN	**98.10%**
MSM-Resnets [56]	20	RGB, D	Im.Net	≈65 M	CNN, LSTM	93.50%
Ahmad et al. [57]	30	RGB	Im.Net	≈150 M	CNN, GRU	91.79%
Tay et al. [58]	-	RGB, OF	HMDB	≈31 M	CNN, LSTM	85.81%
STAN-ARMA [59]	-	RGB, OF	Im.Net	≈34 M	CNN, Att	96.00%
**Ours**	25	RGB, OF	Im.Net	27 M	CNN, Att	**96.60%**

**Notes:** RGB—red–green–blue; OF—optical flow; SMs—saliency maps; D—depth; DIs—dynamic images; Im.Net—ImageNet; K—Kinetics-400 dataset; Sp.1M—Sports-1M dataset; HMDB—HMDB dataset. #F—number of frames used; #Param—number of model parameters. Models are categorized into RNN-based (blue), 2D/3D CNN-based (yellow), and combined deep network approaches (green and coral). Bold values indicate models that achieved high accuracy during evaluation.

Of the 2D/3D CNN-based methods, C3D [33] achieved 85.20% accuracy, while Wang et al. [19] improved it to 89.10% using optical flow. More advanced models such as R(2+1)D + BERT [54] reached 98.60%, but they require more than 100 million parameters and extensive pre-training with more than 30 frames. Our method efficiently selects important frames and achieves high accuracy with fewer resources. For combined deep networks, MSM-ResNets [56] and Bilen et al. [60] reported accuracies of 93.50% and 96.00%, respectively, but they require large models and pre-training. Our approach, which combines ResNet-18, Bi-LSTM, and motion-based frame selection, balances accuracy and efficiency, making it suitable for real-time applications.

### 5.2. Computational Trade-Off Analysis

Our model is designed to select only the most informative frames, which reduces computation without significantly affecting accuracy. As shown in Figure 7, the model reaches around 92% accuracy using fewer than 10 frames, meaning that in many cases only a small number of frames are sufficient to correctly recognize the action. While adding more frames can slightly improve accuracy, exceeding 95% when using more frames, it also increases the computational cost, making the system slower and less efficient. This highlights an important trade-off: fewer frames allow faster and more efficient predictions, which is especially useful for real-time applications or edge devices, while more frames offer only minor accuracy improvements at the cost of higher resource consumption. The model is able to process one frame in 4.5 milliseconds, achieving real-time performance of up to 222 frames per second (FPS). Compared to models that rely on large datasets like Kinetics-400 for pre-training, our approach reaches similar accuracy with lower memory usage and shorter training time due to motion-based frame selection and a lightweight feature extractor. To further test scalability, stress tests were performed by increasing the number of frames per sequence up to 1000. As shown in Table 1, the model exhibited near-linear performance scaling, limited only by the GPU’s 48 GB memory. Additionally, sensitivity tests by varying frame numbers and tuning batch size and sequence length confirmed stable performance, though very large sequences led to increased memory usage and inference time. These findings highlight the model’s robustness and efficiency across different deployment scenarios.

### 5.3. Human–System Interaction and Situational Awareness

**Figure 8:**
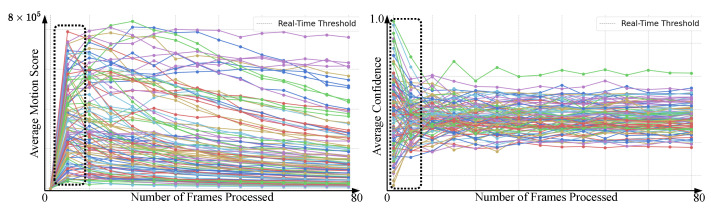
Analysis of motion scores and classification confidence across varying frame intervals for selected videos. The average motion scores of frames and the average confidence of those scores are shown. Different colored lines represent different human action classes.

Our model improves human–system interaction by extracting actionable insights from video data. As shown in Figure 8, high motion scores are captured within approximately 10 frames, allowing classification confidence to be determined with minimal frame processing. This efficiency would allow real-time applications to achieve reliable performance while reducing computational costs.

In surveillance and security, the model should detect suspicious activity early, minimizing unnecessary surveillance and enabling faster response. In healthcare, it can detect falls while reducing false alarms. In human–robot interaction, robots can better anticipate human actions and adapt their behavior, improving safety and collaboration. By focusing on essential frames and filtering out irrelevant ones, the model optimizes decision making and improves situational awareness across domains.

The current model indirectly handles uncertainties through confidence scoring and attention-based feature prioritization. To strengthen reliability, future work will explore explicit uncertainty estimation techniques, allowing the model to quantify its confidence during decision-making, which is especially critical in safety-sensitive applications.

### 5.4. Performance on Multi-Source Actions

Table 3 summarizes the performance of the proposed framework on action recognition tasks using samples collected from multiple sources. These sources include (1) actions from the UCF-50 benchmark dataset [61], (2) video samples retrieved from the internet (links are provided in the public code repository (https://github.com/basheeraltawil/HAR-ResNet-BiLSTM-Attention.git) (accessed on 25 April 2025); this small set of short YouTube clips was selected to introduce more variation and to evaluate the model’s ability to generalize to real-world, unconstrained scenarios); and (3) real-time recordings captured using an Astra RGB camera integrated into a TIAGo mobile robot [12].

**Table 3:** Performance of the framework on multi-source actions.

Input	Ground Truth	Top 3 Predictions (%)	Frame Count	Seq. No.
UCF50:				
lv_Biking_g06_c02.avi	Biking	**Biking (97.9)** CleanAndJerk (0.2) Drumming (0.2)	157	5
v_SalsaSpin_g06_c02.avi	SalsaSpin	**SalsaSpin (93.8)** MoppingFloor (0.5) FrontCrawl (0.4)	167	5
v_SoccerJuggling_g20_c01.avi	SoccerJuggling	**SoccerJuggling (43.9)** TennisSwing (17.7) ThrowDiscus (5.6)	301	10
Internet-Sourced Samples:				
Applymakeup_01	ApplyEyeMakeup	**ApplyEyeMakeup (28.4)** BlowingCandles (11.2) ApplyLipstick (9.6)	450	15
Basketbal_01	Basketball	**Basketball (23.1)** Shotput (15.9) FloorGymnastics (14.0)	479	15
Haircut_01	Haircut	**Haircut (68.0)** Shotput (2.4) BlowDryHair (2.3)	452	15
TIAGo Robot (Real Time):				
Robot Camera	WritingOnBoard	**WritingOnBoard (23.9)** TiaChi (14.4) MoppingFloor (6.3)	4	1
Robot Camera	WallPushups	**WallPushups (30.0)** JumpRobe (11.6) BodyWeightSquats (6.3)	4	1
Robot Camera	Typing	**PlayingPiano (15.7)** Typing (8.6) HammerThrow (8.3)	4	1

The table shows the top 3 predicted actions and their probabilities for different samples from different input sources. The first block contains samples from the UCF-50 [61] dataset, the second block contains samples from the Internet, and the third block shows real-time predictions on the TIAGo robot [12], as shown in Section 4.

As shown in the table, the framework achieved highly accurate predictions for UCF-50 samples, successfully identifying actions such as Biking, SalsaSpin, and SoccerJuggling, all closely matching the ground truth labels with strong confidence scores.

For the internet-sourced videos, the predicted actions (ApplyEyeMakeup, Basketball, and Haircut) were also consistent with their respective ground truth labels, albeit with slightly lower confidence compared to the UCF-50 samples.

**Figure 9:**
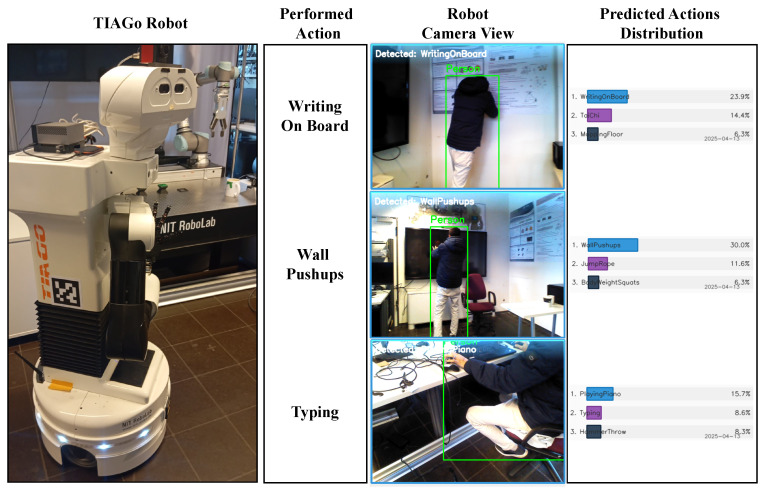
The TIAGo robot [12] engaged in a human action recognition experiment. The left side shows the TIAGo robot observing the environment. The right side shows performed actions, robot camera view, and predicted actions with their distribution percentages. Three example frames are shown, each representing a different human action—wall pushups, writing on a board, and typing.

In the case of real-time experiments with the TIAGo robot, the framework demonstrated reasonable performance under practical conditions. For instance, WritingOnBoard was predicted correctly with approximately 24% confidence and WallPushups with 30%. For the Typing action, the model ranked it as the second most likely prediction, while PlayingPiano was ranked first, likely due to the visual similarity between the two actions, as shown in Figure 9. The figure shows snapshots of the robot’s camera view during the execution of three different actions: WritingOnBoard, WallPushups, and Typing. Alongside each captured frame, the predicted action distribution is visualized, reflecting the model’s confidence levels for the top-ranked actions.

It is important to note that these real-time samples were captured while the robot was both moving and adjusting its head position, introducing challenges such as motion blur, varying viewpoints, and unstable framing. This setup was intentionally used to test the model’s adaptability and to highlight the potential for future improvements in autonomous robotic systems.

Overall, the results confirm that the proposed framework is capable of handling diverse input sources, including benchmark datasets, online videos, and real-time robotic vision, demonstrating robust and transferable performance across different environments.

## 6. Conclusions

In this study, we introduced a deep learning framework for human action recognition (HAR) that balances accuracy and computational efficiency. It uses ResNet-18 for spatial feature extraction, Bi-LSTM for handling time-based information, and a novel frame selection method combining attention mechanisms with motion filtering. This setup allows the model to recognize actions both quickly and accurately, achieving 96.60% accuracy on the UCF-101 dataset and real-time processing of 222 frames per second (FPS). The combination of these components enables the model to effectively learn both short- and long-term dependencies, while the attention mechanism helps guide predictions toward reliable and near-optimal solutions under real-world conditions.

Despite these successes, some challenges remain. The model can struggle to differentiate between actions that are visually similar, such as Typing and PlayingPiano. To improve performance, additional features, such as depth or keypoint information, may be needed.

For future work, we plan to experiment with different datasets, explore alternative backbones beyond ResNet-18, and investigate other types of attention mechanisms. This will help make the model more memory-efficient. To better recognize visually similar actions, we aim to incorporate features like keypoints and depth. Adding uncertainty estimation will enhance the model’s reliability, particularly for safety-critical applications like healthcare and robotics. These improvements will result in a faster, more accurate, and more reliable action recognition system.

## Figures and Tables

**Table 1:** Performance comparison of various configuration parameters.

Frames	Batch Size	Attent. Heads	Hid. Size	Runt. (NM)	Runt. (WM)	Acc. (NM)	Acc. (WM)
20	4	2	500	60	142	86.00%	95.00%
20	8	4	600	55	134	86.69%	**95.80%**
20	16	4	800	52	127	86.30%	95.63%
20	32	8	1000	50	122	86.42%	95.00%
50	4	2	500	120	255	87.16%	95.50%
50	8	4	600	114	248	88.70%	**96.20%**
50	16	4	800	110	245	88.60%	95.60%
50	32	8	1000	107	242	86.60%	95.40%
70	4	2	500	171	320	90.50%	95.44%
70	8	4	600	166	309	91.81%	**96.60%**
70	16	4	800	157	305	91.31%	95.18%
70	32	8	1000	150	300	91.40%	95.31%

**Notes:** Hidden size indicates the size of the hidden layer in the Bi-LSTM model. Runtime (NM) and runtime (WM) represent the time needed for feature extraction and model training without and with motion score in minutes, respectively. Acc. (NM) and Acc. (WM) correspond to the testing accuracy of the model when motion score is either excluded or included, respectively. Bold values and light green rows highlight the best performing results and configuration within each training group.

## Data Availability

No new data were generated in this study.
